# The Correspondence Between Students’ and Teachers’ Views on Teachers’ Emotional Scaffolding Strategies in English Classes in Iran

**DOI:** 10.3389/fpsyg.2022.874747

**Published:** 2022-06-28

**Authors:** Farangis Shahidzade, Ali Mohammad Fazilatfar, Mohammad Hasan Razmi

**Affiliations:** Department of English Language and Literature, Faculty of Languages and Literature, Yazd University, Yazd, Iran

**Keywords:** emotional scaffolding strategies, positive emotions, pedagogy-related emotional scaffolding strategy, behavior-related emotional scaffolding strategy, classroom affects

## Abstract

Academic emotions can play a major role in students’ learning in their English classes. The literature about emotional scaffolding strategies is not widely developed; most of the studies merely focus on negative emotions in EFL contexts. However, in today’s world of varied psychological elements, it is more critical than ever before to scaffold students’ positive emotions to provide more opportunities for their classroom engagement. This study is to explore teachers’ strategies of enhancing students’ positive emotions in English classes in Iran. To investigate the correspondence between students’ and teachers’ views on the effective emotional scaffolding strategies of English teachers, an exploratory sequential mixed-methods design was conducted. First, the researcher conducted semi-structured interviews with 11 high school students of Salami School in Kazeroun to collect in-depth qualitative data. Then, the interview-based Likert scale questionnaires were distributed and completed by 60 high school English teachers in Kazeroun for the quantitative data. According to the teachers’ self-reports, expressing optimism and hope about the students’ future lives and prospective jobs was the most frequently used strategy. There was a significant difference among the teachers’ views on the emotional scaffolding strategies. Moreover, some of the strategies taken into consideration as effective strategies by the students are the least frequently used ones according to the teachers’ reports. The results and findings have several theoretical and practical implications for English teachers in EFL contexts; they might bolster their understanding of appropriate emotional scaffolding strategies preparing them to play a contributing role as great facilitators in English classes.

## Introduction

As Fried (2011) noted, two subsequent movements in psychology, behaviorism and cognitivism, ignored the role of emotions in learning since they were not observable and explicable. Gardner (1987) also highlighted the deliberate feature of cognitive psychology that was the low priority of affective, historical, contextual and cultural factors. According to Rossiter (2003), the development of humanistic psychology as a holistic approach to learners conduced to a growing focus on individuals’ emotions and feelings. Over the past three decades, since the studies in second language acquisition confirmed that attention to learners’ emotions might enhance language learning process, the importance of students’ and teachers’ emotions has been widely highlighted. Linston and Garrison (2003) expressed their hope for more academic concentration on emotions by maintaining the fact that we have kept our emotions at an ontological level to the extent that we turn to them only when a specific problem requires an emotional mode (for example: learner or teacher motivation, job-satisfaction, teacher identity, etc.). Linston and Garrison (2003) believe this conceptualization of emotions is just a small piece of a bigger puzzle. They further explain that such a conceptualization should be revisited:

That seems ill-advised, and it is time to rebuild our academic house. When we teach, we teach with ideas and feelings. When we interact with students, we react and they respond with thoughts and emotions. When we inquire into our natural and social worlds, we do so with desire and yearning (p. 5).

Among the studies focusing on the relationship between classroom affects and second language learning, the majority were non-experimental (Gardner et al., 1987; MacIntyre and Gardner, 1989; Madsen et al., 1991; Price, 1991; Young, 1991; Brown et al., 2001). A number of these studies underlined the importance of implementing emotional scaffolding strategies for early childhood learners due to making contributions to their classroom engagement (Blair, 2002; Hyson, 2008; Park, 2014, 2016). However, concerning the context of secondary school learners, there is little literature on the effective scaffolding strategies that contribute to enhancing students ‘positive emotions in language teaching classes in both teachers’ and students’ views.

Academic emotions as an influential area in language classes especially in EFL contexts require more comprehensive investigation. Considering there has been a multitude of emotion-in-education symposia, edited volumes on emotions and several issues of academic journals highlighting the role of emotions in teaching and learning (Rosiek and Beghetto, 2009), as Dewaele and Li (2018) pointed out, it is time for growing emotional studies through several research methods of quantitative and qualitative designs.

Despite the dramatic changes in the views associated with the importance of emotional factors, it seems likely that positive emotions in language learning situations are not widely investigated in most existing studies, meaning that most of the studies carried out in EFL contexts highlighted the role of negative affective filters; good language learners with a remarkable amount of linguistic achievements are not required to express their positive feelings while learning language (Wilson, 2013). A few studies have been conducted on how teachers can trigger students’ positive emotional responses in language classes (Dewaele, 2015). To the best of the researchers’ knowledge, no specific study has addressed the practical strategies preferred by students to be applied by teachers to scaffold students’ positive emotions in English classes.

## Literature Review

### Emotional Scaffolding Definitions and Components

Emotional scaffolding includes two components. According to Do and Schallert (2004), emotions are the intense, short-term affective conditions of people reacting to particular stimuli. Wood et al. (1976) defined scaffolding as “controlling those elements of the task that are initially beyond the learner’s capacity, thus permitting him to concentrate upon and complete only those elements that are within his range of competence.” To focus on the affective dimension of scaffolding, as Rosiek and Beghetto (2009) noted, emotional was added. Highlighting the linguistic aspect of emotional scaffolding, Rosiek (2003) defined it as “teacher pedagogical use of analogies, metaphors, and narratives to influence students’ emotional response to specific aspects of subject matter in a way that promotes students’ learning” (p. 402).

This research is based on the comprehensive conceptual framework provided by Meyer and Turner (2007) to analyze the strategies teachers use in to emotionally engage language learners in class activities. They defined emotional scaffolding as teachers’ supporting positive emotional experience of learners that leads to enhancing their learning and development. As they noted, its scaffolding component is borrowed from Vygotsky’s zone of proximal development Vygotsky (1978) complemented with positive emotions playing a crucial role in the learning process. The term highlights teachers’ strategies that scaffold students’ emotional engagement in classroom interactions. In their opinion, emotional scaffolding has the unique quality of having clear classroom objectives differentiating it from other forms of teacher–student interactions. They defined it as “Temporary but reliable teacher-initiated interactions that support students’ positive emotional experiences to achieve a variety of classroom goals” (p. 244). Considering the given conditions of applying the strategies within classroom interactions, Meyer and Turner (2007) mentioned that students must be supported temporarily when they urgently need those scaffolding supports. They also highlighted the importance of the strategies in scaffolding the learners to move from those with shared responsibilities to independent ones. Accordingly, the ultimate and interrelated goals of emotional scaffolding are bolstering learners’ understanding, engagement and motivation to raise achievement, competencies, experiences and independence (Meyer and Turner, 2007). More recently, some innovative studies highlight the importance of learners’ emotional engagement with language and task and how these affective factors can practically influence learners’ performances (e.g., Zabihi, 2018; Zabihi et al., 2021; Zabihi and Ghahramanzadeh, 2022).

### The Relationship Between Classroom Affects and Learning

Emphasizing the impacts of students’ emotional states on learning engagement, Park (2014) noted that experiencing negative emotions can lead to decreasing engagement through applying avoidance and disengagement strategies as a means of self-protection whereas positive feelings while taking part in tasks would motivate them to stay engaged and raise their attentiveness to regulate their learning. Regarding the former state, Price (1991) investigated the relationship between linguistic skills, personality types, and language anxiety. The participants were 80 Israelis speaking Hebrew as their mother tongue and learning English as a second language. The results recorded a significant negative correlation between the linguistic skills in both languages and anxiety toward the two languages. Similar levels of anxiety were revealed toward both languages.

Young (1991) also conducted a study on the relationship between anxiety and foreign language proficiency. Sixty participants took the OPI at the University of Texas at Austin, Southwest Texas State University, or the University of Texas at El Paso. They were majors in French, German, or Spanish or prospective French, German, or Spanish teachers. The findings showed significant negative correlations between anxiety and the OPI. Regarding negative emotions, Goleman (1995) noted that “students who are anxious, angry, or depressed do not learn; people who are caught in these states do not take in information efficiently or deal with it well” (p. 78).

Concerning the latter emotional state, other researchers underlined the importance of positive emotions in language learning experiences. According to Young (1990), teachers’ and learners’ classroom interactions can play an important role in creating a supportive and facilitative classroom atmosphere that can reduce foreign language anxiety. Boekaerts (1993) explored the impacts of care and support on classroom achievements. He confirmed the significant role of emotional state in students’ classroom performance by noting that when they are happy with the content in their classroom, their performance will markedly improve.

In the broaden-and-build theory of positive emotions proposed by Fredrickson (2001), positive emotions would build and broaden thought-action repertoire by raising social, psychological and physical resources. The theory takes into account experiencing positive emotions as a fundamental human strength. Schweinle et al. (2006) also acknowledged that the more learning experience is pleasant and the less it is anxiety-producing, the more students attempt to achieve. They analyzed the relationship between motivation and affect. They also explored the relationship between students’ motivation and teachers’ educational practices. The findings revealed that affect plays a vital role in students’ experience of learning.

Moreover, the research conducted by Williams et al. (2013) investigated the relationship between positive emotions of students in the classroom environment and their learning behaviors. The findings revealed a positive relationship between the students’ experiencing positive emotions in the classroom and their motivation as well as their behaviors leading to academic achievements. Dissimilarly, a negative relationship between the experience of positive emotions and emotional exhaustion was also recorded.

In the study carried out by Pavelescu and Petric (2018), the emotional experiences of four adolescent English students in Romania were analyzed. To collect data related to the emotional experiences of learning English inside and outside the class, some qualitative methods were applied. The findings highlighted two predominant positive feelings. The first one, love, contributed to motivating learners in the absence of enjoyment functioning as the second one in some classroom settings. Noteworthy was the distinction observed in the intensity and stability of the participants’ emotions. Two of them represented a strong and stable love of English, while the other two experienced the enjoyment of English language learning. The results also confirmed the dynamic nature of emotions associated with the contexts of language learning.

### Different Studies on Teachers’ Emotional Scaffolding Strategies

According to Patrick and Ryan (2003), teachers play a major role in providing language learners with optimal learning opportunities through psychological scaffoldings. They can elevate students’ educational experiences by encouraging them to express their ideas and feelings freely and critically in discussions. Teachers can develop a supportive atmosphere for students so that they can feel positive; this positive feeling can make an important contribution to their concentration and willingness to participate in classroom practices. Teacher’s supports in the classroom would encourage the students’ task-related interactions and the use of self-regulated learning strategies.

There has been a multitude of studies highlighting the relationship between specific teachers’ strategies and positive emotions. According to Schweinle et al. (2006), giving positive feedback and suggestions for improvement would provide a supporting atmosphere of acknowledging students’ competence. The supporting opportunities for success can enhance students’ accomplishments in non-competitive ways. In other words, teachers would keep their students excited, hopeful and determined to learn more through applying the psychologically scaffolding strategies. As Williams et al. (2013) pointed out the strategies applied by successful teachers like effective communication skills, eagerness and empathy can result in students’ more engagement and achievements.

The qualitative case study by Park (2016) explored scaffolding emotions applied by two early childhood teachers in a public elementary school. The data collection instruments were observations, interviews, and documents. Based on their beliefs about self-identities and self-interpretation, the teachers applied different strategies to support their students with appropriate emotional scaffoldings. The findings demonstrated how the teachers’ beliefs, knowledge, and other contextual factors contributed to constructing emotional scaffolding strategies. In terms of teachers’ understanding of their students’ emotional feelings and applying corresponding emotional strategies, the teachers implemented their academic knowledge of how young children learn generally, how the specific group of students learns, how they learn as individuals, and how the students give emotional responses in their classes. This study revealed that emotional scaffolding functions as a critical pedagogical means helping teachers approach developmentally appropriate activities conducive to academic success. However, the number of research describing the strategies teachers apply to scaffold their students emotionally in classrooms is limited (Meyer and Turner, 2007). Meyer and Turner (2007) enlisted different scaffolding approaches teachers take to initiate and sustain trust such as expressing their expectations explicitly, making eye contact with students, narrating personal events, modeling enthusiasm and enjoyment while taking part in interactions, using the sense of humor and complimenting students’ strength.

## Research Objectives

Providing a positive classroom climate that would scaffold motivation, self-regulation, collaboration and positive emotions of different language learners with varying levels of competence and needs is complicated (Meyer and Turner, 2002). The purpose of this study is to investigate the correspondence between students’ and teachers’ perspectives about the effective emotional scaffolding strategies that English teachers implement in their English classes to raise their students’ positive emotions associated with language learning practices.

More specifically, the following research questions guided this study:

1. In high school students’ views, what are the teachers’ scaffolding strategies for enhancing students ‘positive emotions in language teaching classes in Iran?
2. In high school teachers’ views, what are the effective teachers’ scaffolding strategies for enhancing students ‘positive emotions in language teaching classes in Iran?
3. Is there any correspondence between the effective emotional scaffolding strategies applied by English teachers in students’ and teachers’ views?

## Methodology

### Participants

#### Senior High School English Learners

As it was noted (Bernard, 2017), implementing purposive sampling in qualitative research can supply rich information through selecting the communicative subjects willing to convey their experiences. Accordingly, purposive sampling was applied to select the student interviewees. They consisted of 11 students of Salami High school in Kazeroun. All of them were high school students with an average age of 18. Their school has been the best public high school in the city. They were selected as the most practical samples due to their interest and regular engagement in English classroom interactions.

#### Senior High School English Teachers

Among the 75 English teachers of high school in Kazeroon, 60 of them including both males and females responded to the questionnaires given to them. They were M.A or B.A holders. Their age range was between 25 and 40 years and their experience in teaching English in different high schools ranged from 3 to 18 years. Their selection was based on convenience sampling.

### Instrumentation

The data collection instruments were teacher questionnaires and student interviews. To collect in-depth qualitative data sets, the researchers conducted semi-structured interviews. To expand and enrich the study, the inquirers gathered the data through semi-structured interviews with 11 students. The interviews encompassed open-ended questions and discussions. Besides being a helpful method of gathering the respondents’ invisible views and experiences on English teachers’ strategies fostering psychological emotions in language learners, the interviews also provided ample opportunities for the learners to vocalize their authentic and deep perspectives in terms of the strategies supporting their positive emotions. Conducting open-ended interviews contributed to detecting the hidden angle of the students’ personalities and preferences.

An interview-based Likert scale questionnaire (Appendix) was administered to the teachers under study to collect the quantitative data. The questionnaire was constructed by changing the coded qualitative data of semi-structured interviews into the Likert items of the questionnaire. It was conducted to explore whether the data collected through the interviews corresponded with the results from the questionnaires. Scrutinizing the questionnaire items helped the researchers to code all items as the two main categories of pedagogy and behavior-related strategies of teachers applicable in classes. The pedagogical items were items No. 3, 9, 10, 11, and 15 including the strategies directly associated with language teaching procedures such as using films, songs and music in teaching English while items No. 1, 2, 4, 5, 6, 7, 8, 12, 13, 14, 16, 17, 18, 19, 20 were considered as positive behavior-related strategies applied by teachers in English classes such as having eye contact with students in the class.

### Design of the Study

An exploratory sequential mixed-methods design was applied to conduct multi-dimensional research to investigate the effective emotional scaffolding strategies of English teachers in teachers’ and students’ views. First, it involved a semi-structured interview administered to 11 selected high school students. While analyzing the data, the team of researchers worked collectively to devise some specific categories and themes. The emerged themes helped in uncovering and clarifying the descriptions and outcomes of effective emotional strategies of teachers thoroughly. Then, based on the qualitative data, a Likert-scale questionnaire was devised to collect the quantitative data. To recapitulate, the researcher used the quantitative and qualitative data in a complementary way to get more valid, detailed and in-depth views of both stakeholders.

### Data Collection and Analysis Procedures

The team of researchers collected the qualitative data by conducting semi-structured interviews with 11 students of Salami High school in Kazeroun who were consented to participate in the interview. Student respondents were asked to reflect on their positive emotional experiences where teachers played the role of emotional scaffolders in their English classes. They were also required to recall and express the effective strategies their teachers used to raise their students’ positive emotions. The researcher allocated half an hour to each interviewee to respond to the questions and provide more details and clarifications. Their answers were transcribed and coded by hand to obtain more valid, deep and rich descriptions of emotional scaffolding strategies of teachers. By content-analyzing, the researchers explored the relationship between different concepts emerged and developed two main categories of teachers’ emotional scaffolding strategies with a sufficient level of saturation.

To collect the quantitative data of the teacher participants, an interview-based Likert-scale questionnaire was constructed. To calculate internal consistency, the questionnaires were piloted for 10 high school teachers. The Cronbach’s alpha-coefficient calculated was promising (Cronbach’s α = 0.72). To ensure the relevance of the questionnaire items to the construct under study, three professors of applied linguistics were asked to fill in the questionnaire with 24 items. They were asked to determine whether these items are relevant in terms of the teachers’ emotional scaffolding strategies applicable in English classes. They had to rate each item based on a Likert scale ranging from 1 standing for irrelevant to 4 which stands for extremely relevant. They were also asked to write some comments about the questionnaire items. The experts’ comments contributed to reconstructing, eliminating and rewording some items. Accordingly, the final questionnaire included 20 revised and relevant items. Regarding the questionnaire items, the researcher classified all items into pedagogical and positive behavioral strategies of teachers. Items No. 3, 9, 10, 11, and 15 were accounted as pedagogical strategies while items No. 1, 2, 4, 5, 6, 7, 8, 12, 13, 14, 16, 17, 18, 19, 20 were classified as positive behavioral ones.

Then, the questionnaires were responded by the 60 high school English teachers teaching English in Kazeroun in Iran. Teacher respondents were asked to fill in the Likert-scale questionnaire to represent their views on the appropriate emotional scaffolding strategies they apply in their English classes to facilitate classroom interactions and lead to language acquisition and achievements. Teachers had to self-report the frequency of applying each emotional scaffolding strategy entitled as never, sometimes, often and always.

One of the researchers explained the purpose and significance of the study to all participants to elicit valid and relevant qualitative and quantitative data. Similar explanations about the technical terms used in the questionnaire and interview were also provided for all participants. The researcher mentioned that their participation was voluntary and their responses would remain confidential.

## Results and Findings

### The Emotional Scaffolding Strategies of Teachers in the Student’ Views

Student 1: When my English teacher is eager to teach, I feel eager to learn. The teacher’s enthusiasm for teaching English can motivate and energize me more to continue performing the demanding task of language learning. My enthusiastic teacher works as a model for me to make more efforts to learn English more efficiently. Most of the time, energetic teachers are informative because they do their best to be competent teachers. When my teacher is happy and hopeful while teaching, I am motivated to listen to her and answer her questions. Teaching English is a tough task but my English teacher should do her best to control her negative emotions. In my opinion, an emotional supporter should have a balanced state of mind if she intends to be a real model for us. Teachers themselves should feel positive to create an emotionally supportive classroom environment for us.

Student 2: Understanding the fact that my English teacher is knowledgeable and skillful in English teaching can assure me that I can learn English efficiently just like my teacher. I feel excited when I see that my English teacher can deal with challenges successfully in her class. Her fluent Speaking and accurate pronunciations make me more interested in such an amazing language class. When I see that my teacher knows a lot about the second language, I am motivated.

Student 3: Using some computer-assisted activities in language teaching can insert positive emotion into our setting. I would like to learn English by different technological devices to add variety to the language learning conditions. Besides, using films, songs and music in teaching English will add variety to our routine class activities.

Student 4: I mostly feel happy when my English teacher laughs and has fun with us in class. Sometimes, her positive eye contact and smiley face while telling her life stories reduce my stress. Calling my first name or even nickname when asking a question also represents friendly relationships among the class members. Raising friendly relationships among my classmates in class can provide a supportive, not a competitive setting where I trust others and ask for help when needed. This supportive atmosphere can help me learn and perform more successfully in my English class.

Student 5: Sharing the relevant personal real-life experience with us in English class represents that English is not just the language of the textbook but applicable to real-life settings. I am eager to learn English to communicate with others through a foreign language. Seeing its practicality in real life can improve my positive feeling conducive to more achievements due to enhancing life literacy. Moreover, I have a strong sense of belonging and attachment to the language classroom community when the teacher provides chances for us to express our positive and negative feelings through classroom discussions. As an effective member of the community whose emotional state is important, I make efforts to improve my state emotionally and academically.

Student 6: When my English teacher engages me in different classroom tasks, whether challenging or non-challenging, I feel that I can be an active learning member of the classroom community. Most of the time, encouraging us to initiate discussions in class can make me feel worthy due to the appreciation of my critical thinking in the English class. Allocating specific time to express our personal stories while listening to her real-life narratives can help us to develop both linguistically and mentally. It is crucial to have an expressive and friendly teacher if I am going to express my own experiences.

Student 7: While taking part in class activities, I expect my English teacher to reward me in line with my progress and improvement. If she does so, I feel positive. Regardless of my marks and proficiency level, when the teacher appreciates my efforts, I am psychologically supported. I feel relaxed when my teacher evaluates my competence in a non-comparative and non-competitive setting focusing on my gradual development and progress.

Student 8: Treating all my classmates equally respectful regardless of our English proficiency level raises my positive feelings in English classes. A fair teacher can excite me by rewarding both weak and strong students according to their immediate class activities and improvements not the teachers’ background knowledge of their educational status.

Student 9: Allowing us to take part in pair work/group work activities in class helps us learn effectively from each other due to mutual trust and contribution. I also experience a sense of responsibility in the group. The teacher should allow us to manage our team activities. She should not regularly control our group work. When my team can do the task independently without asking the teacher for help, I feel proud.

Student 10: Giving corrective feedback to me without disrespecting me makes me truly listen to what she suggests. I can understand what she explains and I can get the point. Because of understanding her positive intention, I feel positive. The teacher should not repeat my wrong structure or vocabulary a lot. It makes me ashamed in front of my classmates. She should try to help me by emphasizing what I have learned not to impede my learning by highlighting my mistakes. The teacher can help me not demotivate me in the initial steps of learning. I think that I can gradually help myself by using my richer linguistic resources to be a good language learner.

Student 11: Raising optimism and hope in me can assist me to make more efforts. Fostering positive views about my future life, prospective job and practical language skills can help me to tackle the challenges I meet while learning English in class. Due to my positive views, I dare to take part in several demanding but rewarding tasks of language acquisition.

In sum, the emotional scaffolding strategies preferred by student participants are represented in Table 1.

**Table 1:** Teachers’ emotional scaffolding strategies in the student participants views.

Emerging Strategies	Subsequent Outcomes
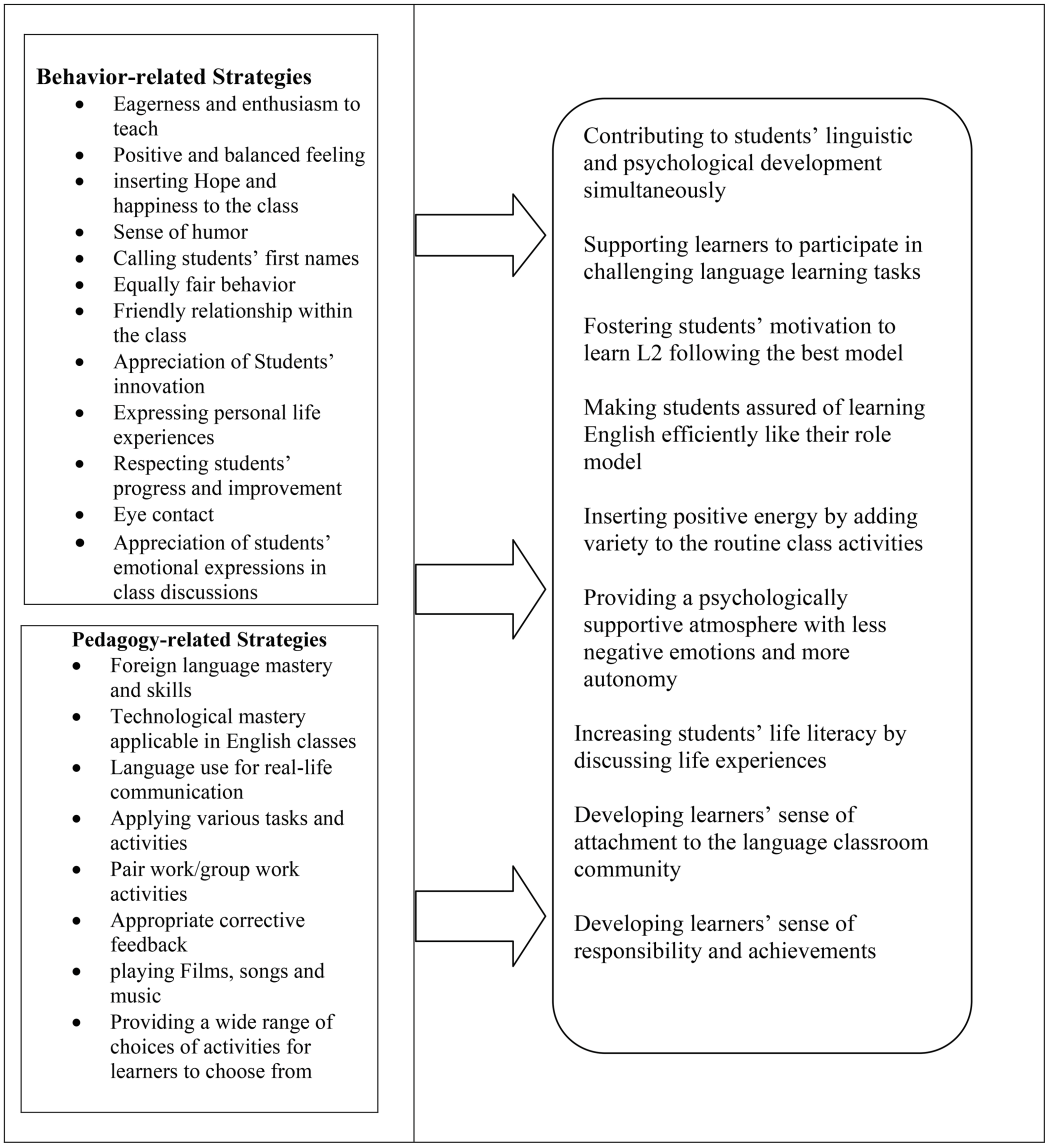

### The Teachers’ Views on Emotional Scaffolding Strategies

Sixty high school English teachers teaching English in Kazeroun responded to the questionnaires. They reported the frequency of emotional scaffolding strategies they use. According to Friedman Test conducted, it was revealed that expressing optimism and hope about students’ future lives and prospective jobs (No.18) was ranked as the most frequently used strategy and having positive eye contact with students (No.14) was the second one. Sharing the relevant real-life experience with students in English classes (No.13) was taken into consideration as the least frequently used strategy according to the teachers’ self-reports.

Considering the questionnaire items responded by the teachers (Table 2), there is a significant difference among the teachers’ views on their effective emotional scaffolding strategies applied in classes (Table 3), because the null hypothesis has been rejected (*p* < 0.01).

**Table 2:** The ranks of emotional scaffolding strategies in the teachers’ views.

Item number	Mean	Rank
Q18	13.04	1
Q14	12.83	2
Q4	12.79	3
Q6	12.79	4
Q12	12.39	5
Q7	12.16	6
Q1	11.71	7
Q17	11.55	8
Q19	11.51	9
Q5	11.18	10
Q16	10.70	11
Q10	10.47	12
Q20	9.76	13
Q9	9.55	14
Q8	8.87	15
Q3	8.34	16
Q11	8.02	17
Q15	7.75	18
Q2	7.58	19
Q13	7.02	20

**Table 3:** Friedman test statistics of the emotional scaffolding strategies in teachers’ views.

N	60
Chi-square	160.935
Df	19
Asymp. Sig.	0.000

### Pedagogy-Related Emotional Scaffolding Strategies of the Questionnaire Items

As the questionnaire indicated, items No. 3, 9, 10, 11, and 15 were the strategies directly associated with language teaching, pedagogy (Table 4). To investigate the ranks of these strategies in terms of the frequency of occurrence, a Friedman Test was conducted (Table 5). The teachers ranked different strategies as follows:

**Table 4:** The ranks of pedagogy-related emotional scaffolding strategies in the teachers’ views.

Item number	Mean	Rank
3.48	Q10	1
3.19	Q9	2
2.84	Q3	3
2.79	Q11	4
2.70	Q15	5

**Table 5:** Friedman test statistics of the pedagogy-related emotional scaffolding strategies in the teachers’ views.

N	60
Chi-square	13.963
Df	4
Asymp. Sig.	0.007

According to Friedman Test administered, providing students with the opportunity to take part in pair work/group work activities in class (No. 10) was rated as the most frequently used pedagogical strategy of teachers enhancing students’ positive emotions while using some computer-assisted activities in language teaching (No. 15) was ranked as the least-frequently used according to the teachers’ self-reports.

In terms of the questionnaire items associated with the pedagogy-related strategies, there is a significant difference among the teachers’ views about these five items since the null hypothesis has been rejected (*p* < 0.01).

### Behavior-Related Emotional Scaffolding Strategies of the Questionnaire Items

Regarding the questionnaire items, the items 1, 2, 4, 5, 6, 7, 8, 12, 13, 14, 16, 17, 18, 19, and 20 were considered as behavioral strategies applied by teachers in English classes (Table 6).

**Table 6:** The ranks of behavior-related emotional scaffolding strategies in the teachers’ views.

Item number	Mean	Rank
Q18	9.57	1
Q14	9.32	2
Q4	9.31	3
Q6	9.23	4
Q12	9.05	5
Q7	8.79	6
Q1	8.53	7
Q19	8.37	8
Q17	8.34	9
Q5	8.03	10
Q16	7.67	11
Q20	7.03	12
Q8	6.35	13
Q2	5.31	14
Q13	5.10	15

Friedman Test administered (Table 7) revealed that expressing optimism and hope about students’ future lives and prospective jobs (No. 18) was ranked as the first frequently used item of behavior-related strategies by the teachers whereas sharing the relevant personal and real-life experience with students in English classes (No. 13) was the least frequently applied.

**Table 7:** Friedman test statistics of the behavior-related emotional scaffolding strategies in the teachers’ views.

N	60
Chi-square	113.402
Df	14
Asymp. Sig.	0.000

A significant difference was observed among the teachers’ views on these 10 strategies because the null hypothesis was rejected (*p* < 0.01).

### Behavior- and Pedagogy-Related Emotional Scaffolding Strategies

To investigate the difference between the overall behavior and pedagogy-related emotional scaffolding strategies in the teachers’ views, the Mann–Whitney *U*-test was performed. Tables 8 and 9 show the results as follows:

**Table 8:** The Mann–Whitney *U*-test statistics of the behavior and pedagogy-related emotional scaffolding strategies.

Mann–Whitney *U*	105423.000
Wilcoxon W	150573.000
Z	−6.067
Asymp. Sig. (two-tailed)	0.000

**Table 9:** The ranks of behavior and pedagogy-related emotional scaffolding strategies in the teachers’ views.

The mean of rank	Emotional scaffolding strategy	Rank
633.36	Behavioral	1
501.91	Instructional	2

The Mann–Whitney *U*-test illustrated a significant difference between the teachers’ views on the overall behavior- and pedagogy-related emotional scaffolding strategies since the null hypothesis was rejected (*p* < 0.01).

The test also indicated that overall behavior-related strategy was ranked as the first effective one functioning as emotional scaffolding whereas overall pedagogy-related one was the second one. In other words, the teachers use the former more frequently than the latter to foster their students’ positive emotions.

## Discussion and Conclusion

As a newcomer to the second language acquisition field, emotions have drawn a lot of attention in SLA research. The role of emotions in language teaching and learning has been investigated by some quantitative, qualitative and mixed-method designs. In the current educational situation of Iran, developing an empirically based theory for scaffolding the emotional feelings of students in their stressful English classes seems vital. Going beyond a reliance on teachers’ intuitions, a relevant grounded theory can empower the teachers to provide their students with appropriate affective opportunities to cultivate their positive emotions. This cultivation can facilitate learners’ active engagement in classroom activities and subsequent achievements (Schweinle et al., 2006).

This study explored teachers’ effective emotional scaffolding strategies in teachers’ and learners’ views. To the researcher’s knowledge, it is the first study analyzing the correspondence between English teachers’ and learners’ views associated with effective emotional scaffolding strategies implemented in classroom interactions in Iran. A sequential mixed-method design was applied to investigate the correspondence between students’ and teachers’ views on the strategies. The qualitative data were collected by semi-structured interviews with 11 purposefully selected students. To collect the quantitative data, an interview-based Likert scale questionnaire was applied. Sixty high school English teachers responded to the questionnaires.

Regarding the first research question, the students mentioned various strategies that could be applied by teachers to enhance students ‘positive emotions in language teaching classes in Iran. The researchers categorized the emotional scaffolding strategies they preferred into pedagogy and behavior-related ones. While the former highlighted the implicit function of instruction-related strategies in scaffolding learners’ emotions, the latter emphasized the explicit role of some positive behaviors in fostering emotional scaffolding. As the outcomes of applying these strategies in language classes, the student participants enlisted increasing students’ participation, motivation, autonomy, linguistic and psychological development, life skills, sense of belonging to the classroom community and further achievements. Among the outcomes reported by the students, increasing learners’ independence, motivation, engagement, psychological and linguistic growth and gradual achievements were congruent with the results recorded by Meyer and Turner (2007). It is also worth mentioning that fostering learners’ motivation, active participation and interaction in classroom activities corresponds with the common results revealed by the previous studies on the impacts of applying emotional scaffolding strategies in language learning contexts (Patrick and Ryan, 2003; Williams et al., 2013; Park, 2014; Ziashahabi et al., 2020).

In terms of the second research question, in teachers’ views, expressing optimism and hope about students’ prospective lives and jobs is the first ranked strategy, and having eye contact with students is the second one. Considering the teachers’ views about the emotional scaffolding strategies applied by English teachers in classes, a significant difference is revealed. The results also indicate that providing opportunities for the high school learners to take part in pair work/group work activities in class is the most frequently used pedagogy-related strategy fostering emotional scaffolding whereas expressing optimism and hope about students’ future lives and prospective jobs is the behavior-related item applied more frequently than other behavior-related ones. Noteworthy is a significant difference between the ranks of overall behavior and pedagogy-related strategies in teachers’ views representing that the teachers would likely prefer behavioral strategies to scaffold their students emotionally rather than the pedagogical ones.

Turning to the third question, some of the emotional scaffolding strategies accounted as effective strategies by the student interviewees are the least frequently used strategies according to the teachers’ self-reports; they include teachers’ sharing relevant personal real-life experiences with students in English classes and using some computer-assisted activities in language teaching as the pedagogical strategy. Approximately a quarter of the student participants (28%) underlined the importance of the expressive atmosphere of language classes where both teachers and students are willing to verbalize and regulate their emotions. However, according to the statistics, the item related to this specific strategy was rated as the least frequently used one by the teachers in question. This inconsistency might be attributed to the teacher-oriented attitudes of the teachers considering themselves as the leading authorities responsible for imparting their academic knowledge to their passive students; teachers are accounted as the principal figures in classrooms not the influential members of class communities providing opportunities for mutual communication of emotional feelings in an atmosphere of trust. Therefore, it is advisable to raise awareness among different stakeholders in language learning contexts, be it teachers or learners, about language learners’ and teachers’ rights to communicate their feelings in an effectively expressive way.

## Pedagogical Implications, Limitations, and Further Suggestions

The findings of this study have several theoretical and practical implications for English teachers. Firstly, language teachers’ perceptions of diverse emotional scaffolding strategies could be clarified and expanded. Secondly, the results of this study would likely be important in pre-service and in-service teacher education programs in Iran in order to be translated into practice based on the cultural teaching-learning contexts; teachers can have valuable guidelines to conduct their instructional and class management activities in accordance with the strategies preferred by students to associate the students’ complicated experiences of language learning with the scaffolding positive feelings. Besides, as professional teachers with the results in mind, they might attempt to develop and internalize appropriate scaffolding strategies to reconstruct the English classroom atmosphere by directing the learners’ emotions positively.

Moreover, the results will likely bolster the English teachers’ understanding of the emotional strategies preferred by the students in the language learning context in Iran. This would empower the teachers with pedagogical knowledge about the effective cultural-congruent ways of scaffolding emotions, preferred by both language teachers and learners, to increase learners’ participation and engagement in classroom activities.

Regarding the nature of sampling applied in this investigation, the generalizability of findings would be limited. Noteworthy is that socio-cultural contexts impact the students’ and teachers’ views on the emotional scaffolding strategies applicable in English classes (Zembylas, 2003). Good strategies would likely be related to cultural values, beliefs and norms. Other researchers can replicate this research by investigating the selected emotional scaffolding strategies in different cultures to understand the commonalities as well as the diversities. They can also investigate the relationship between socio-cultural patterns such as gender and the priorities by learners of different genders. Further studies could be conducted in different classroom contexts with larger samples of English teachers and learners to construct the contextualized theory of effective emotional scaffolding strategies making an important contribution to unifying language teaching and learning.

## Data Availability Statement

The raw data supporting the conclusions of this article will be made available by the authors, without undue reservation.

## Ethics Statement

The studies involving human participants were reviewed and approved by Foreign Languages Department, Yazd University, Iran. The patients/participants provided their written informed consent to participate in this study.

## Author Contributions

All authors listed have made a substantial, direct, and intellectual contribution to the work and approved it for publication.

## Conflict of Interest

The authors declare that the research was conducted in the absence of any commercial or financial relationships that could be construed as a potential conflict of interest.

## Publisher’s Note

All claims expressed in this article are solely those of the authors and do not necessarily represent those of their affiliated organizations, or those of the publisher, the editors and the reviewers. Any product that may be evaluated in this article, or claim that may be made by its manufacturer, is not guaranteed or endorsed by the publisher.

## Appendix

Questionnaire: For each one of the items, please place just one checkmark in the row that is in line with your view. This is not a test and there is no right or wrong answer. Thank you very much for your cooperation.

**Table:**

Personal Background						
Years of teaching:	Age:	Gender				
How frequently do you use each strategy in your class?			Always	Often	Some times	Never
Calling your students’ first name in class Laughing and having fun with your students in class Using films and, songs and music in teaching English Treating your students equally with respect regardless of their English proficiency level Raising friendly relationships among the classmates in class Showing enthusiasm for teaching English in class Appreciating your students’ efforts not just their grades Rewarding your students in accordance with their progress and improvement Involving your students in challenging tasks in English classes Giving your students the opportunity to take part in pair work/ group work activities in class Letting your students make a choice while taking part in different classroom activities Appreciating your students’ creativity and innovation in English class Sharing your relevant personal real-life experience with your students in English class Having positive eye contact with your students Using some computer-assisted activities in language teaching Doing your best to control your negative emotions in class. Giving corrective feedback to your students without disrespecting them Expressing Optimism and hope about your students’ future lives and prospective jobs Starting discussion in class to let your students express their emotions freely Letting them talk about their personal relevant stories in class						
